# Dynamic Patterns of Global Brain Communication Differentiate Conscious From Unconscious Patients After Severe Brain Injury

**DOI:** 10.3389/fnsys.2021.625919

**Published:** 2021-09-09

**Authors:** Daniel Golkowski, Rebecca Willnecker, Jennifer Rösler, Andreas Ranft, Gerhard Schneider, Denis Jordan, Rüdiger Ilg

**Affiliations:** ^1^Department of Neurology, School of Medicine, Technical University of Munich, Munich, Germany; ^2^Neurologische Klinik und Poliklinik, University of Heidelberg, Heidelberg, Germany; ^3^Department of Anesthesiology and Intensive Care, School of Medicine, Technical University of Munich, Munich, Germany; ^4^Asklepios Clinic, Department of Neurology, Bad Tölz, Germany

**Keywords:** consciousness, brain injury, coma, unresponsive wakefulness syndrome, fMRI, anesthesia, propofol, sevoflurane

## Abstract

The neurophysiology of the subjective sensation of being conscious is elusive; therefore, it remains controversial how consciousness can be recognized in patients who are not responsive but seemingly awake. During general anesthesia, a model for the transition between consciousness and unconsciousness, specific covariance matrices between the activity of brain regions that we call patterns of global brain communication reliably disappear when people lose consciousness. This functional magnetic imaging study investigates how patterns of global brain communication relate to consciousness and unconsciousness in a heterogeneous sample during general anesthesia and after brain injury. First, we describe specific patterns of global brain communication during wakefulness that disappear during propofol (*n* = 11) and sevoflurane (*n* = 14) general anesthesia. Second, we search for these patterns in a cohort of unresponsive wakeful patients (*n* = 18) and unmatched healthy controls (*n* = 20) in order to evaluate their potential use in clinical practice. We found that patterns of global brain communication characterized by high covariance in sensory and motor areas or low overall covariance and their dynamic change were strictly associated with intact consciousness in this cohort. In addition, we show that the occurrence of these two patterns is significantly related to activity within the frontoparietal network of the brain, a network known to play a crucial role in conscious perception. We propose that this approach potentially recognizes consciousness in the clinical routine setting.

## Introduction

From a basic research perspective, the subjective experience of being aware of oneself and the environment most likely emerges as an epiphenomenon of cerebral information processing (Dehaene et al., 2003, 2017) and is thus encoded in the activity of the brain and its neurons. Attempts to identify brain activity specific for state of being consciously focused on the question of where conscious perception takes place (Dehaene and Changeux, 2011) and how brain regions are affected by models for unconsciousness, namely, general anesthesia, sleep, or unconsciousness after brain injury (Brown et al., 2010). These approaches were able to link conscious brain function with widespread brain networks: the frontoparietal network, default mode network, and ascending reticular activation system as modulators of these (Boveroux et al., 2010; Demertzi et al., 2015; Bonhomme et al., 2016; Ranft et al., 2016). The activity within these networks could be associated with conscious perception [frontoparietal network, Dehaene et al. (2003) and He et al. (2007)], mind wandering [default mode network, Vanhaudenhuyse et al. (2011)], or the level of consciousness (Gili et al., 2013). In addition, the activity within these networks was specifically diminished during general anesthesia irrespective of the anesthetic agent used (Boveroux et al., 2010; Jordan et al., 2013; Bonhomme et al., 2016; Ranft et al., 2016). However, measuring the activity only in the named networks in patients after brain injury only unreliably detected intact consciousness after brain injury (Vanhaudenhuyse et al., 2010). We hypothesized that conscious processing in the brain requires not only specific networks as a common endpoint but also involves various levels of information processing.

Based on this idea, our data analysis aimed to integrate different levels of hierarchy and considered information processing in the human brain to be divided into (Dehaene et al., 2017) local information processing within specialized brain areas, for example in the primary visual cortex, and (Dehaene et al., 2003) global communication reflected by information exchange between such areas, for instance when we react to complex external stimuli. Both types of information processing potentially play an important role in the generation of consciousness (Barttfeld et al., 2015; Ranft et al., 2016; Demertzi et al., 2019; Golkowski et al., 2019). In addition, we assumed that the dynamics of this local and global information processing is a key feature of consciousness.

Local information processing in unconscious humans is known to be significantly diminished in the medial prefrontal cortex, precuneus, posterior cingulate cortex, superior parietal lobe, and in the dorsolateral prefrontal and inferior parietal cortices during general anesthesia when analyzed by functional MRI (fMRI) (Ranft et al., 2016; Golkowski et al., 2019). In awake humans, these brain areas frequently communicate and are known as the default mode network and the frontoparietal network. This data analysis identified this local information processing through independent maps of a spatial independent component analysis’ (ICA) resulting in 57 brain regions covering the whole brain and their time course of activity.

Global communication has been mainly investigated between specific brain areas using fMRI and EEG. Like local information processing, differences between consciousness and unconsciousness were observed mainly in the frontoparietal network and the default mode network. Specifically, information exchange between the areas encompassing these networks was significantly reduced in various studies on unconsciousness (He et al., 2007; Jordan et al., 2013; Bonhomme et al., 2016; Ranft et al., 2016; van Vugt et al., 2018). Later, it was demonstrated that patterns of global brain communication are significantly altered during unconsciousness and that the transition between patterns was reduced (Barttfeld et al., 2015; Hudetz et al., 2015; Ma et al., 2017; Golkowski et al., 2019). Similar results were obtained in patients suffering from disorders of consciousness after brain injury, thus showing that the concept of global brain communication can be generalized to patients with different reasons for unconsciousness (Luppi et al., 2019). We modeled this global brain communication by calculating the correlation between brain regions in a sliding window approach, resulting in a series of correlation matrices.

**FIGURE 1:**
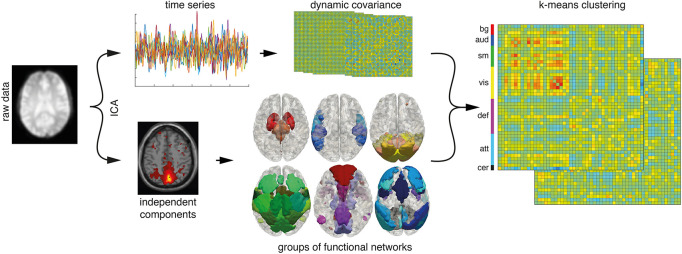
Data analysis pipeline. An independent component analysis (ICA) separates the preprocessed signal into 75-time series (top row) and associated spatially independent components (bottom row). Time courses are sorted into functional groups. Two hundred sixty-seven dynamic covariance matrices are calculated from the time courses of signal components (*n* = 57) in 30 frames time windows. K-means clustering sorts the matrices into seven patterns or clusters. bg, basal ganglia; aud, auditory; sm, somatosensory and motor; vis, visual; def, default mode; att, attentional; cer, cerebellar networks.

We reasoned that specific patterns of global brain communication and their dynamic change are an absolute requirement for the emergence of consciousness. In order to identify specific patterns of global brain communication, we employed a k-means algorithm on covariance matrices. The change of covariance matrices over time was regarded as the dynamics of global brain communication (Figure 1).

## Materials and Methods

### Ethics Statement

The study was conducted in accordance with the Declaration of Helsinki, and the protocol of the study was approved by the ethics committee of the medical school of the Technische Universität München (München, Germany). The volunteers or the legal surrogates of the enrolled patients were given detailed information about the procedures and potential risks prior to written informed consent. Medical history was taken from every applicant, especially inquiring contraindications for an examination in a magnetic resonance scanner. Data were acquired between April 2013 and December 2019.

### Participants

Eighteen patients (eight male and 10 female, aged 36–84 years) were acquired through intensive care units. Inclusion criteria were patients with unresponsive wakefulness syndrome (UWS) through any brain damage, i.e., traumatic (*n* = 3), stroke (*n* = 13), anoxia (*n* = 1), and toxic/metabolic (*n* = 1). Under stroke, we summarized ischemic stroke as well as subarachnoid hemorrhage and intracerebral hemorrhage with no traumatic cause. We included all types of brain injuries for two main reasons: (1) we wanted to generalize the approach, and (2) we aimed to define the AWAKE state by specific brain activity and the UWS state by the absence of this activity. Based on this, we regarded the exact brain pathology as secondary. Any preexisting medication was left unchanged for the measurement accepting oral sedatives (e.g., low-dose benzodiazepines or neuroleptics). Intravenous sedatives were not applied. The subjects fasted for 6 h prior to the measurement. The patients with UWS were rated using the Coma Recovery Scale Revisited (CRS-R) at least three times (the day before the measurement, on the day of measurement, and in one follow-up) by an experienced medical doctor. All the patients had UWS on the day of measurement. All the other volunteers [*n*(propofol) = 11, *n*(sevoflurane) = 14, *n*(controls) = 20] were healthy, male volunteers (aged 20–36 years) and had no contraindication for general anesthesia using propofol or sevoflurane (American Society of Anesthesiology I). All the subjects with contraindications for MRI or positron emission tomography (PET) were excluded. We included unmatched healthy controls for the patients with UWS recorded on the same PET scanner that was also used for the UWS experiments. Because of the complex nature of the experiment (e.g., application of a PET contrast agent), we were unable to record a group of age-matched patients with cerebral pathology during the AWAKE state. This approach might introduce a bias since group differences might be affected by structural brain alterations or age differences.

### Experimental Protocol

#### Controls and UWS Patients

For AWAKE and UWS resting-state measurement, the participants lay supine in the scanner and were told not to fall asleep while keeping their eyes closed.

#### Propofol Anesthesia

The first resting-state recording was acquired identical to the control subjects. In the following, this condition is referred to as AWAKE. The measurement during propofol-induced loss of responsiveness (PROP) was carried out using a target-controlled infusion pump (Open TCI; Space infusion system; Braun Medical, Melsungen, Germany). Propofol concentration was increased by 0.4 μg/ml steps beginning at 1.2 μg/ml until volunteers stopped responding to the verbal command “squeeze my hand” (equivalent to a Ramsay sedation scale score of 5–6). The concentration was then kept stable for the remaining fMRI measurement. This point was reached at plasma concentrations of 2.97 +/− 0.47 μg/ml (mean +/− SD). Ten minutes of equilibration time were waited before the actual measurement took place. Throughout this article, this state is referred to as PROP. Details of the propofol anesthesia protocol can be found in Jordan et al. (2013).

#### Sevoflurane Anesthesia

The resting-state was acquired identical to the propofol setting. Image acquisition during the sevoflurane-induced loss of responsiveness (in this article: SEVO) was carried out after intubation with a magnetic resonance tomograph-compatible laryngeal mask and during artificial ventilation using an anesthesia machine (Fabius Tiro, Dräger, Germany). Sevoflurane was kept stable at 2 volume percent end-tidal concentration during this condition. The subjects were unresponsive to the command “squeeze my hand” during this condition, tolerated the laryngeal mask well, and showed reduced movements when compared with the wakeful state. It is also noteworthy that clinically, this sedation was deeper when compared with PROP (corresponding to a Ramsay scale score of 6). All the participants were asked for any memories of the unresponsive state, and all reported amnesia for the procedure. Details of the sevoflurane anesthesia protocol can be found in Ranft et al. (2016).

### Data Acquisition

Data from the patients with UWS (*n* = 18) and healthy controls (*n* = 20) were acquired using an integrated Siemens Biograph mMR scanner (Siemens Medical Solutions, Erlangen, Germany) capable of registering concurrent positron emission tomography (PET) and MRI from 3 T data using the vendor-supplied 12-channel phase-array head coil. PET volume, two T2^∗^-weighted echo-planar imaging (EPI) MRI, and magnetization-prepared rapid acquisition gradient echo (MPRAGE) T1-weighted anatomic volume data were recorded. Scanning parameters of the EPI included repetition time (TR)/echo time (TE)/flip angle, 2,000 ms/30 ms/90°; 35 slices (gap 0.6 mm) aligned to the anterior-posterior commissure covering the whole brain; field of view (FoV), 192 mm; matrix size, 64 × 64; and voxel size, 3 mm × 3 mm × 3.0 mm. Each of the two measurements consisted of 300 acquisitions in interleaved mode, with a total scan time of 10 min and 8 s. Scanning parameters for the MPRAGE sequence included TR/TE/flip angle, 2,300 ms/2.98 ms/9°; 160 slices (gap, 0.5 mm) covering the whole brain; FoV, 256 mm; matrix size, 256 × 256; and voxel size, 1 mm × 1 mm × 1 mm. The total scan time was 5 min and 3 s. The PET data were not further included in this study.

Both the propofol and sevoflurane image acquisitions were carried out using a 3-T whole-body magnetic resonance tomographic scanner (Achieva Quasar Dual 3.0T 16CH; Philips, Medical Systems International Inc., Best, Netherlands) employing an eight-channel, phased-array head coil. fMRI was performed with a gradient EPI sequence (echo time = 30 ms, repetition time = 1.838 ms, flip angle = 75°, field of view = 220 mm × 220 mm, matrix = 72 × 72, 32 slices, slice thickness = 3 mm, and 1 mm interslice gap; 300 volumes were acquired in the propofol cohort and 350 volumes were acquired in the sevoflurane cohort. Of these 350 frames, the last 50 were discarded). Anatomy was acquired before the functional scan using a single T1-weighted sequence and 1 mm × 1 mm × 1 mm voxel size per subject. Only data sets that not exceeded 2 mm of translation in either z-, y-, or z-direction were included in the subsequent data analysis.

### fMRI Data Analysis

DPARSF 4.0 (Chao-Gan and Yu-Feng, 2010) and SPM12 (Friston, 2007) were used for preprocessing. Functional and anatomical images were realigned manually along the AC-PC plane. The first three time points were removed, and slice timing was corrected. Images were segmented and normalized using DARTEL to a voxel size of 2 mm × 2 mm × 2 mm. Functional images were smoothed with a 4 mm full-width at half-maximum Gaussian kernel. A 2 mm × 2 mm × 2 mm-voxel-size template was created from a standard epi template in SPM12 (“EPI.nii”). Both the anatomical and functional images were co-registered to this template. Six movement parameters (x-, y-, and z-translation and the corresponding rotations) and their first derivatives were regressed out using a general linear model. The time series were de-spiked after the regression and band-pass filtered between 0.01 and.1 Hz. No other global signal removal or scrubbing of the frame was performed.

In the first step, the preprocessed fMRI data were analyzed using a group-level spatial iICA (applied on the whole data set, namely AWAKE, PROP, SEVO, and UWS) as implemented in the Gift toolbox (version 4.0b, Group ICA/IVA of fMRI Toolbox; Georgia State University, Atlanta, GA, United States) and GICA3 back reconstruction. The INFOMAX algorithm was employed together with an ICASSO and 20 repetitions to decompose the data into 75 spatially independent components. We used the GICA3 back reconstruction to generate individual spatial maps and associated (potentially correlated) time courses for each individual map (Figure 1). We chose ICA because it was able to identify networks in altered brain anatomy robustly, and it represents the standard technique of dimensionality reduction/brain parcellation in altered states of consciousness [see, e.g., Demertzi et al. (2014)].

This resulted in two types of data: (1) spatially independent components for each subject that were z-standardized. We refer to these maps as S_*i*_; and (2) one time course for each component and each subject. The time courses were also standardized to be standard normally distributed to account for different scanners and amplitudes of the raw blood oxygenation level-dependent signal. We refer to these time courses as R_*i*_.

A signal from neuronal origin was assumed if the component projected on gray matter showed no similarity to venous vessels and if it showed a characteristic frequency spectrum with a clear peak below 0.1 Hz. Noise components usually showed a flat frequency spectrum similar to Gaussian noise. Fifty-seven functional networks were identified and included in the subsequent analysis. These networks were close to identical to the ones previously published (Golkowski et al., 2019).

The R_*i*_ time courses were despiked and low-pass filtered < 0.15 Hz (Allen et al., 2014; Golkowski et al., 2019). Correlation matrices were calculated in a sliding window manner as implemented in the Temporal dFNC toolbox of GIFT 4.0b using 30-time points from each measurement, resulting in 267 correlation matrices per measurement (which is 300 recorded volumes minus 3 discarded during preprocessing minus sliding window length). The size of the resulting matrices was 57 × 57 due to the symmetry of the correlation matrices they contained 57 × 56/2 independent data points. To make the correlation matrices more accessible for visual inspection, we sorted the functional networks into groups, namely, basal ganglia networks, auditory networks, somatosensory and motor networks, visual networks, default mode networks, attentional networks, and cerebellar networks. It is noteworthy that this sorting into functional groups has no effect on the later k-means clustering algorithm since the algorithms only use the L1 distance of the correlation matrices (i.e., the sum of the absolute differences) as a measure of distance and is, therefore, independent of the order of correlation values.

We used the k-means clustering algorithm also implemented in GIFT 4.0b with the L1-(“Manhattan”)-distance with 20 repetitions to assign the between-network connectivity matrices to between network-connectivity patterns. These patterns are not predefined and are generated by the k-means algorithm as clusters or patterns of the between-network connectivity matrices. The generally arbitrary number of patterns was defined to be 7 to make the results comparable to Allen et al. (2014) and Golkowski et al. (2019).

After variable ranking using the Fisher score, one to seven variables were chosen for a support vector machine. The aim was to test whether the information from the anesthesia experiments can be generalized. For training of the support vector machine algorithm, the 50 data sets from the anesthesia experiments were employed. Eighteen data sets from the patients with UWS and 20 controls were used for the test data set. We also conducted the support vector machine algorithm with a random permutation of groups of the test data set to illustrate chance level. These steps were carried out with FSLib (Version 5.1). All analysis steps were carried out in Matlab (R2016a; Mathworks, Natick, MA, United States).

### Statistical Analysis

All the group comparisons were conducted with the two-sided Mann–Whitney/rank sum test. *P*-values were corrected for multiple comparisons using Bonferroni’s method (multiplication factor 6). Differences in pattern distribution between the groups (on the nominal scale of pattern numbers) were tested by using a chi-square test with Pearson’s chi-square distance. Significance was assumed if *p* < 0.05.

Logistic regression was carried out in Matlab using the “mnrfit” and logit transfer functions. The binary categorical responses of the appearance of patterns specific for consciousness (patterns 1 and 3) and all the other patterns were modeled on the time series of each individual network (*n* = 57) after smoothing using the mean of 30 frames sliding window. This was the same slide window length from which the covariance matrices were calculated. The time series were transformed to be standard normally distributed in each recording to remove variance or amplitude effects. Significance was assumed if *p* < 0.05.

## Results

### Baseline Characteristics

Aiming to transfer findings from the model of anesthesia-induced unconsciousness to disorders of consciousness, we divided the data into a training data set, namely, subjects during wakefulness and general anesthesia, and a test data set, namely, patients with UWS and healthy controls. The training data set undergoing general anesthesia comprised 11 healthy volunteers during wakefulness and propofol-induced general anesthesia (PROP) and of 14 healthy volunteers during wakefulness and sevoflurane-induced general anesthesia (SEVO). The test data set comprised 20 healthy volunteers and 18 patients with UWS (see Table 1 for baseline characteristics). The one patient with 9 points in the CRS-R fulfilled no criteria for being minimally conscious on the day of measurement but showed eye fixation the day before. We decided to include this patient because the measurement had a sufficient data quality, and we saw a higher chance to find patterns specific for intact consciousness in this patient. We do not think that this introduced a bias to data analysis. Four patients were more than 100 days after brain injury, and 14 patients were included in the study during the acute hospitalization with less than 42 days after brain injury.

**TABLE 1:** Clinical features of the unresponsive, wakeful patients.

	Mean ± SD	Range
Age, y	62 ± 14	36–84
Time since injury, d	62 ± 60	18–206
CRS-R on day of measurement	4.1 ± 1.9	2–9
Sex	10 female	8 male
Etiology, number		
Stroke	13	
TBI	3	
Anoxia	1	
Metabolic	1	

### Pattern Separation

**FIGURE 2:**
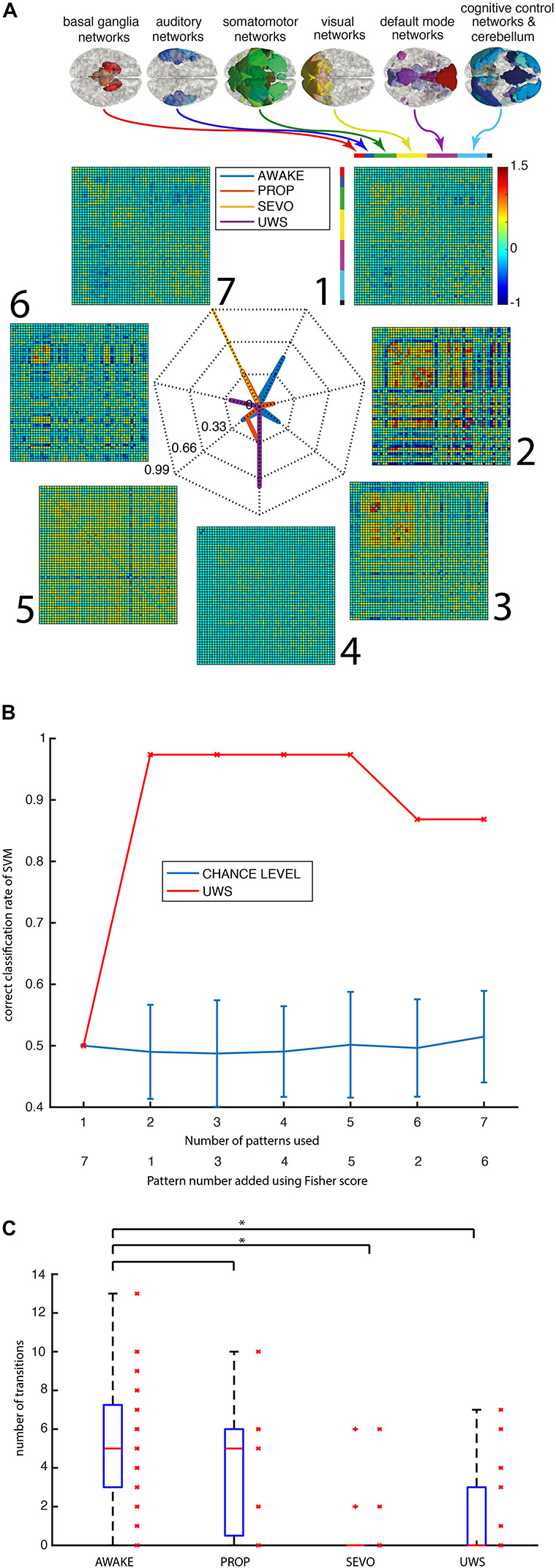
Patterns are specific for intact consciousness. **(A)** Individual data points of the pattern matrices arise from different groups of functional networks: basal ganglia, auditory, somatomotor, visual, default mode, cognitive control, and cerebellar networks. The radar plot shows the patterns 1–7 and their relative distribution of appearance in the different groups (AWAKE in blue, PROP in red, SEVO in yellow, and UWS in purple). **(B)** Correct classification rate of a support vector machine trained in anesthesia data and tested on the subjects with UWS (*n* = 19) and 19 controls (red) vs. chance level in a random assignment of groups and 100 repetitions (blue with standard errors). **(C)** An absolute number of transitions between patterns in the four groups. (*p < 0.05, Mann–Whitney test, uncorrected).

To test if patterns of global brain communication are specific for consciousness, we conducted an ICA with 75 components using the complete data set and separated functional brain networks (*n* = 57) from noise sources (*n* = 18). For each network, we then calculated the associated covariance matrices in 30-frame time window each (Figure 1). This resulted in 267 covariance matrices per recording. The k-means clustering of all the resulting matrices into seven clusters showed that the various groups had a significantly different distribution across distinct patterns of global communication (*p* < 0.001 for AWAKE vs. PROP, SEVO and UWS, chi2test, uncorrected, Figure 2A). Patterns 1 and 3 were almost exclusively assumed by the AWAKE subjects (probability: pattern 1:0.5, pattern 3:0.22) and rarely during PROP (probability: pattern 1:0.015, pattern 3:0.001). Patterns 1 and 3 did not occur in UWS and SEVO subjects. The distribution of patterns showed an overlap between the SEVO group, which was almost exclusive in pattern 7 (probability 0.99), and the PROP group (probability for pattern 7:0.37). The latter group also showed an overlap with AWAKE subjects (mainly pattern 5) and subjects with UWS (pattern 4). Pattern 2 was only observed during PROP (probability 0.13), and pattern 6 exclusively appeared in the UWS (probability 0.26) group. In summary, this clustering showed that patterns 1 and 3 are specific for the AWAKE group.

To test if the appearance of specific connectivity patterns is sufficient to distinguish consciousness from unconsciousness, we separated the data into one training data set containing the subjects from PROP (*n* = 11), SEVO (*n* = 14), and AWAKE (*n* = 25) and one test data set containing the UWS (*n* = 18) and AWAKE subjects from the same MRI scanner (*n* = 20, these subjects were not included in the training data set). We classified the test data set employing a support vector machine and prior Fisher scoring for feature selection (Figure 2B). We composed the groups this way because general anesthesia, unlike many types of disorders of consciousness, is a well-controlled, reversible form of unconsciousness with intact brain anatomy. As expected from the k-means clustering, the inclusion of only one pattern (pattern 7) resulted in a chance level result, because this pattern was only encountered in the SEVO and PROP groups. Combining patterns 1 and 7 resulted in a correct classification rate of 0.97. The one misclassified data set was from the AWAKE group. Precision remained constant upon further addition of features and then declined above 6 features. We concluded that knowledge about the subjects being either in the PROP, SEVO, or AWAKE group allowed a precise prediction of whether patients were in the UWS or the AWAKE group.

### Pattern Dynamics

Next, we tested if decreased dynamics of connectivity patterns are a feature of diminished consciousness (Figure 2C) by analyzing the absolute number of transitions between the different patterns of global brain communication as the parameter. The difference was significant for SEVO and UWS vs. controls (*p* < 0.05, Mann–Whitney test, uncorrected) but not for PROP vs. controls. We also observed a relevant overlap between the groups and the span of values in the AWAKE group was (0, 13), thus covering the whole range of encountered values in all the groups. Consequently, the dynamics of global brain communication could not be used as a specific predictor of consciousness.

**FIGURE 3:**
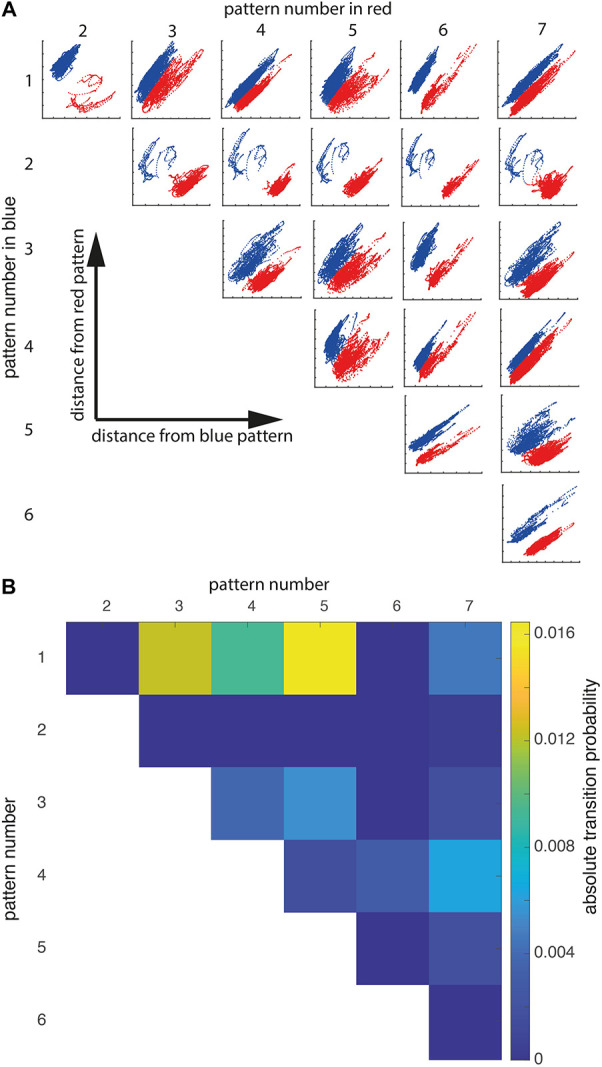
State transitions are continuous and specific. **(A)** Pairwise plots showing how k-means clustering separated the dynamic covariance matrices into patterns. The blue pattern number is given in the left-side column, the red pattern number is in the top row. The x-axis gives the distance from the blue pattern, while the y-axis gives the distance from the red pattern. For example, the top left plot shows covariance matrices classified as pattern 2 in red and covariance matrices classified as pattern 1 in blue. The x-coordinate of a given data point is the distance from pattern 1, whereas the y-coordinate is given by distance from pattern 2. Units are omitted since they are arbitrary. **(B)** Matrix showing the absolute transition probability from pattern X (top row) to Y (left-sided column) or vice versa.

The distribution of patterns of global brain communication across groups and their dynamics had two further implications: (1) the AWAKE subjects can be both in patterns specific for AWAKE and patterns that can also be seen in the other groups, and (2) the absolute number of transitions is not specific for AWAKE, while the ability to transition to certain patterns is. To better understand when these group-specific transitions happen, we considered L1 distances (the sum of the absolute difference) of single covariance matrices with respect to a given pattern. Since the resulting space had seven dimensions (one dimension for each distance of covariance matrix to one of the seven clusters), we visualized the data in a pairwise manner (Figure 3A). This resulted in 21 plots, giving the L1 distances of covariance matrices from a pair of patterns and the pattern they were classified as. This visualization demonstrated that there are different types of separations into patterns: patterns separated visually by a gap and patterns with a continuous transition. For example, the AWAKE-specific pattern 1 shows a continuous transition with patterns 3, 4, and 5. In contrast, pattern 2 only exhibits this type of transition with pattern 7, two patterns that were only encountered during general anesthesia. In summary, the plots show how subjects within a group move rather continuously from one pattern to another than in a binary manner.

This observation was further supported by the transition probability matrix (Figure 3B). The probability to change between patterns of global brain communication that show a gap in Figure 3A was zero or close to zero, while pattern separation without gap showed frequent transitions. Notably, patterns observed in the AWAKE group showed the highest probability of transitions. Hence, the absolute number of transitions was not a characteristic for consciousness but the ability to transition into specific patterns of global brain communication.

**FIGURE 4:**
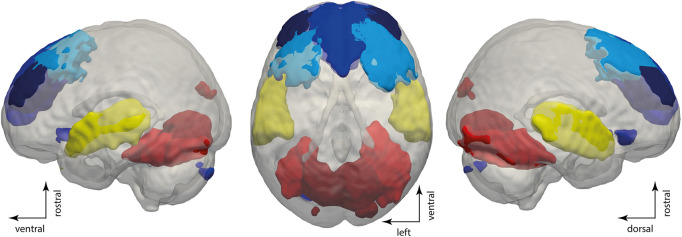
Specific network activity is related to the appearance of conscious-related patterns. Rendering showing the dorsolateral and dorsomedial prefrontal cortices in blue, primary auditory cortices in yellow, and visual cortices in red. The rendering shows maps of the independent components with a threshold of *p* < 0.05 family-wise error correction in SPM12.

Aiming to identify networks driving these patterns associated with consciousness, we conducted a logistic regression with patterns associated with consciousness, namely patterns 1 and 3, as the response variable and the time courses of all networks as predictors. This regression showed that 6 out of 56 networks had a significant positive slope with respect to the appearance of conscious-related patterns. These networks encompassed sensory networks: the bilateral primary auditory cortex (*p* < 0.05, *B* = 0.31), the bilateral calcarine gyrus (*p* < 0.05, *B* = 0.28), and the bilateral fusiform gyrus (*p* < 0.05, *B* = 0.32), as well as networks of the frontal lobe: the bilateral dorsomedial prefrontal cortex (*p* < 0.05, *B* = 0.37), the bilateral dorsolateral prefrontal cortex 1 (*p* < 0.001, *B* = 0.49), and the bilateral dorsolateral prefrontal cortex 2 (*p* < 0.05, *B* = 0.33, see Figure 4). Thus, primary auditory and visual cortices as well as dorsolateral and dorsomedial prefrontal areas’ activity is positively related to the appearance of patterns associated with consciousness.

## Discussion

In summary, our data analysis demonstrates that patterns of global brain communication specific for consciousness exist in the model of general anesthesia, irrespective of anesthetic agent. The patterns specific for AWAKE were characterized by either a high correlation across various hierarchical levels of sensory and motor systems (pattern 3) or by a state of low overall correlation (pattern 1). This finding could be translated to patients during unresponsive wakefulness and controls, where the same patterns reliably identified intact consciousness and were absent in UWS. One main difference between the model of general anesthesia and patients with UWS is certainly the altered brain anatomy. The UWS cohort, however, also included subjects with intact brain anatomy and only subcortical pathology. The findings were also applicable to these individuals. In addition, the ICA is largely independent of the brain anatomy and was able to identify brain networks outside of lesioned brain tissue. We regard this relative independence of the data analysis pipeline from anatomical alterations as an advantage over atlas-based brain parcellations, allowing the future automatization of this approach. In summary, we hope that the direct identification of conscious information processing might be superior when compared with current state-of-the-art approaches using signal complexity (Casarotto et al., 2016) or machine learning of large data sets of various states of consciousness (Demertzi et al., 2015) in the future. Theoretically, the direct measurement of conscious-specific brain activity might be more sensitive toward intact consciousness than surrogate markers that are statistically linked to the clinical state.

Both the models of general anesthesia and UWS showed reduced dynamics between patterns when compared with the AWAKE state. However, we encountered a relevant overlap between the different groups. This result demonstrates that the AWAKE state is much more characterized by continuous transitions between patterns specific and not specific for AWAKE than by the mere number of transitions. How such patterns arise in the brain has been investigated by computational models showing that specific patterns and their dynamics arise from the connectome of the human brain only if the brain works at a “critical” level (Haimovici et al., 2013; Deco et al., 2017), a tight balance between excitation and inhibition. In addition, both focal lesioning (Alstott et al., 2009; Gratton et al., 2012) and pharmacological manipulation (Tagliazucchi et al., 2016; Lee et al., 2019) were predicted to lead the activity of the brain away from this level. These theoretical considerations also gained experimental support in animals (Ma et al., 2017) and humans during general anesthesia with different anesthetic agents (Zhang et al., 2018; Golkowski et al., 2019). Our data illustrate that both specific patterns and their dynamic change are abolished during unconsciousness irrespective of the exact cause. Thus, this study is in line with these theoretical properties of brain organization and previous experiments further bridging the gap between theory and the subjective experience of being conscious.

The activity within the dorsal prefrontal cortical areas, primary sensory networks, was also linked to the appearance conscious-related patterns of global brain communication. This observation is not only a mere reproduction of previous findings on the role of the dorsal attention system in consciousness (Ranft et al., 2016; Golkowski et al., 2019) but also highlights that the activity in the dorsal attention system (Mashour et al., 2020) is temporally related to patterns of global brain communication specifically for the AWAKE state in both the model general anesthesia and in the clinical setting.

### Limitations of the Study

In our study, the control cohort for the test data set consisted of healthy volunteers. The separation of the two groups is presumably easier than between a group of wakeful patients with severely damaged brains and our UWS group, a setting in clinical practice where this approach has to prove whether it actually delivers additional information to the clinical evaluation, i.e., is able to identify intact consciousness in patients with UWS in a real-world setting.

In addition, the presented data analysis is very complex and not yet fully automatized. For an application in clinical routine, the analysis pipeline needs to be implemented in an automatized toolbox and made available to the general public.

The limited number of subjects in each group is certainly another limiting factor. For the anesthesia experiments, it was already difficult to obtain ethics committee approval for a limited number of subjects. For the patients with UWS, it took several years to obtain sufficient data sets of adequate quality in this single-center study.

These two major flaws should be addressed in a future study equipped with an easily available analysis tool and a comprehensive clinical study design.

### Conclusion

In summary, the findings of this study show that complex patterns of global brain communication are capable to separate consciousness from unconsciousness during both general anesthesia and disorders of consciousness. Thus, this approach might not only reveal intact consciousness in seemingly wakeful but unresponsive patients but also give an insight into the neurophysiological basis of consciousness itself.

## Data Availability Statement

The raw data supporting the conclusions of this article will be made available by the authors, upon reasonable request.

## Ethics Statement

The studies involving human participants were reviewed and approved by Ethics committee of the Technical University Munich. The patients/participants provided their written informed consent to participate in this study.

## Author Contributions

DG designed and conducted the experiments, conducted the data analysis, and wrote the manuscript. RW and JR conducted the experiments. AR, DJ, and RI designed and conducted the experiments and helped in writing the manuscript. GS helped in writing the manuscript. All authors contributed to the article and approved the submitted version.

## Conflict of Interest

The authors declare that the research was conducted in the absence of any commercial or financial relationships that could be construed as a potential conflict of interest.

## Publisher’s Note

All claims expressed in this article are solely those of the authors and do not necessarily represent those of their affiliated organizations, or those of the publisher, the editors and the reviewers. Any product that may be evaluated in this article, or claim that may be made by its manufacturer, is not guaranteed or endorsed by the publisher.
